# Sirtuin 2 inhibitor AGK2 exerts antiviral effects by inducing epigenetic suppression of hepatitis B virus covalently closed circular DNA through recruitment of repressive histone lysine methyltransferases and reduction of cccDNA

**DOI:** 10.3389/fcimb.2025.1537929

**Published:** 2025-04-09

**Authors:** Jumi Kim, Jiseon Ha, Chanho Song, Muhammad Azhar Sajjad, Fadia Kalsoom, Hyeonjoong Kwon, Jaewoo Park, Sun Park, Kyongmin Kim

**Affiliations:** ^1^ Department of Microbiology, Ajou University School of Medicine, Suwon, Republic of Korea; ^2^ Department of Biomedical Science, Graduate School of Ajou University, Suwon, Republic of Korea

**Keywords:** hepatitis B virus, covalently closed circular DNA, SIRT2 inhibitor AGK2, repressive histone lysine methyltransferases, epigenetic suppression

## Abstract

Chronic hepatitis B virus (HBV) infection continues to be a global health concern because current treatments such as interferon-α and nucleos(t)ide analogs cannot fully eliminate the virus due to persistence of covalently closed circular DNA (cccDNA) and integrated HBV DNA. Earlier research suggests that AGK2, a selective SIRT2 inhibitor, suppresses HBV replication by modifying key signaling pathways. This study aimed to further explore the anti-HBV effects of AKG2, particularly its effects on the epigenetic landscape of cccDNA. HBV-transfected and -infected cells were used to assess the impact of AGK2 on viral replication. Changes in SIRT2 expression and α-tubulin acetylation (SDS-PAGE-immunoblotting), core particle formation (native agarose gel electrophoresis and immunoblotting), HBV RNA (northern blotting) and DNA (Southern blotting) synthesis, and cccDNA levels (Southern blotting) were measured. Chromatin immunoprecipitation assays were performed to examine deposition of transcriptionally repressive epigenetic markers on cccDNA. AGK2 reduced expression of SIRT2, increased acetylated α-tubulin levels, and reduced synthesis of HBV RNA and DNA. Importantly, AGK2 also reduced cccDNA levels and increased deposition of repressive histone markers H4K20me1, H3K27me3, and H3K9me3 on cccDNA, mediated by histone lysine methyltransferases such as PR-Set7, EZH2, SETDB1, and SUV39H1. Additionally, there was a reduction in recruitment of RNA polymerase II and acetylated H3 to cccDNA, indicating that AGK2 enhances transcriptional repression. AGK2 suppresses HBV replication through direct antiviral actions, and by epigenetic modulation of cccDNA, indicating that using AGK2 to target SIRT2 and associated epigenetic regulators shows promise as a functional cure for chronic hepatitis B.

## Background

Hepatitis B virus (HBV) infection remains a global public health concern due to significant morbidity and mortality, even though a highly effective vaccine is available (Dusheiko et al., 2023; Jeng et al., 2023). According to the World Health Organization (WHO), 296 million people worldwide are living with HBV infection, and 820,000 people died in 2019 (Jeng et al., 2023). HBV causes acute and chronic hepatitis B (CHB), which can progress to fibrosis, cirrhosis, or hepatocellular carcinoma (Dusheiko et al., 2023; Jeng et al., 2023). To date, two types of antiviral drug, interferon-α (IFN-α) and nucleos(t)ide analogs, are approved for CHB treatment (Dusheiko et al., 2023; Jeng et al., 2023). Although these drugs suppress viral replication, neither eliminate the HBV covalently closed circular DNA (cccDNA) minichromosome; only elimination is considered a complete cure for HBV (Martinez et al., 2021; Dusheiko et al., 2023). The HBV cccDNA minichromosome, a transcriptional template, comprises both histone and non-histone host and viral proteins (Levrero et al., 2009; Martinez et al., 2021; Dusheiko et al., 2023), resulting in a chromatin-like structure that encapsulates nuclear transcription factors and chromatin remodeling enzymes within the cell nucleus (Pollicino et al., 2006; Saeed et al., 2019; Piracha et al., 2020; Martinez et al., 2021; Saeed et al., 2021; Locatelli et al., 2022). Epigenetic modifications on the cccDNA minichromosome during HBV infection are crucial for modulating viral transcriptional activity (Belloni et al., 2009; Levrero et al., 2009; Belloni et al., 2012; Dandri, 2020; Piracha et al., 2020; Martinez et al., 2021; Locatelli et al., 2022). These changes make a significant contribution to disease pathogenesis and development of associated cancers (Belloni et al., 2009, 2012; Dandri, 2020; Martinez et al., 2021; Locatelli et al., 2022).

Sirtuins (SIRTs) belong to atypical class III histone deacetylase family that cleaves acetyl and/or acyl groups from lysine within histones or other substrates in a manner dependent on the cofactor nicotinamide adenine dinucleotide (Carafa et al., 2016; Alqarni et al., 2021; Wu et al., 2022). Seven mammalian SIRT proteins have been identified (Carafa et al., 2016; Alqarni et al., 2021; Wu et al., 2022), all of which play roles in regulating multiple cellular processes such as transcriptional activity, survival and proliferation, apoptosis, autophagy, mitochondrial energy homeostasis, metabolism, DNA repair, inflammation, and oxidative stress responses (Carafa et al., 2016; Alqarni et al., 2021; Wu et al., 2022). SIRTs are involved in numerous metabolic and regulatory processes during infections by human immunodeficiency virus, influenza A virus, herpes simplex virus 1, coronavirus, and human papillomavirus (Budayeva et al., 2016; Alqarni et al., 2021; Silva et al., 2023). SIRTs are broad-spectrum, evolutionarily conserved factors that defend against viruses (Koyuncu et al., 2014; Budayeva et al., 2016). SIRT2, SIRT3, SIRT6, and SIRT7 interact with HBV cccDNA and inhibit viral transcription through epigenetic modifications (Ren et al., 2018; Piracha et al., 2020; Kong et al., 2021; Yu et al., 2021; Yuan et al., 2021). Targeting of SIRTs to regulate epigenetic modifications that suppress HBV transcription may be an attractive approach to curing HBV infection.

SIRT2 is known to mediate histone deacetylation at H4K16ac, H3K18ac, and H3K56ac (Vaquero et al., 2006; Das et al., 2009; Eskandarian et al., 2013) In addition, SIRT2 is a bona fide tubulin deacetylase, responsible for catalyzing the deacetylation of acetylated α-tubulin at K40 (North et al., 2003). In our previous study, we demonstrated that overexpression of SIRT2 isoform 1 (SIRT2.1) or SIRT2 isoform 5 (SIRT2.5) increases or decreases cccDNA levels, respectively (Piracha et al., 2020). Moreover, we found that SIRT2.5 is recruited to cccDNA to a greater extent than SIRT2.1. Overexpression of SIRT2.5 increases recruitment of PR-Set7, enhancer of zeste homolog 2 (EZH2), suppressor of variegation 3–9 homolog 1 (SUV39H1), and SET domain bifurcated 1 (SETDB1) (Piracha et al., 2020). Conversely, overexpression of SIRT2.1 decreases recruitment of these same histone lysine methyltransferases (HKMTs). Increased recruitment of repressive HKMTs enhances deposition of transcriptional repressive epigenetic markers such as monomethyl-H4K20 (H4K20me1), trimethyl-H3K27 (H3K27me3), and trimethyl-H3K9 (H3K9me3) (Piracha et al., 2020).

Recent studies have identified SIRT modulators that are effective against inflammation, cancer, metabolic diseases, neurodegenerative diseases such as Parkinson’s disease, and viral infections (Carafa et al., 2016; Hackett et al., 2019; Yang et al., 2020b; Alqarni et al., 2021; Wu et al., 2022; Lombardo et al., 2024). Among them, several small molecules inhibit HBV replication (Piracha et al., 2018; Yu et al., 2018; Jiang et al., 2019; Kong et al., 2021; Roche et al., 2023; Tang et al., 2024). For example, the selective SIRT2 inhibitor AGK2 (2-Cyano-3-[5-(2,5-dichlorophenyl)-2- furanyl]-N-5-quinolinyl-2-propenamide) exerts anti-HBV activity (Piracha et al., 2018; Yu et al., 2018), possibly by downregulating AKT/GSK-3β/β-catenin signaling (Piracha et al., 2018).

In this study we investigated the anti-HBV mechanisms employed by AGK2 (other than repression of AKT/GSK-3β/β-catenin signaling) (Piracha et al., 2018). Consistent with previous reports from ourselves and others, we found that AGK2 treatment decreased HBV replication (Piracha et al., 2018; Yu et al., 2018) by reducing SIRT2 expression and increasing that of acetylated α-tubulin (Piracha et al., 2018). Moreover, AGK2 treatment resulted in a significant reduction in HBV covalently closed circular DNA (cccDNA), leading to the decreased syntheses of HBV RNA and replication intermediate (RI) DNAs. To further elucidate the mechanisms by which AGK2 inhibits transcription of HBV we conducted chromatin immunoprecipitation (ChIP) assays, which revealed that AGK2 increased deposition of repressive epigenetic markers (H4K20me1, H3K27me3, and H3K9me3) via the activity of heterochromatin-linked HKMTs such as PR-Set7, EZH2, SETDB1, and SUV39H1. Additionally, AGK2 decreased recruitment of RNA polymerase II and acetylated H3 to cccDNA, further contributing to transcriptional repression of HBV. This and previous (Piracha et al., 2018) findings indicate that AGK2 exerts multifaceted antiviral effects on the HBV life cycle by modulating SIRT2 expression, α-tubulin acetylation, and epigenetic modification of cccDNA. Understanding these molecular mechanisms may provide novel therapeutic strategies for targeting HBV infection.

## Results

### HBV replication increases endogenous SIRT2, resulting deacetylation of α-tubulin

**Figure 1:**
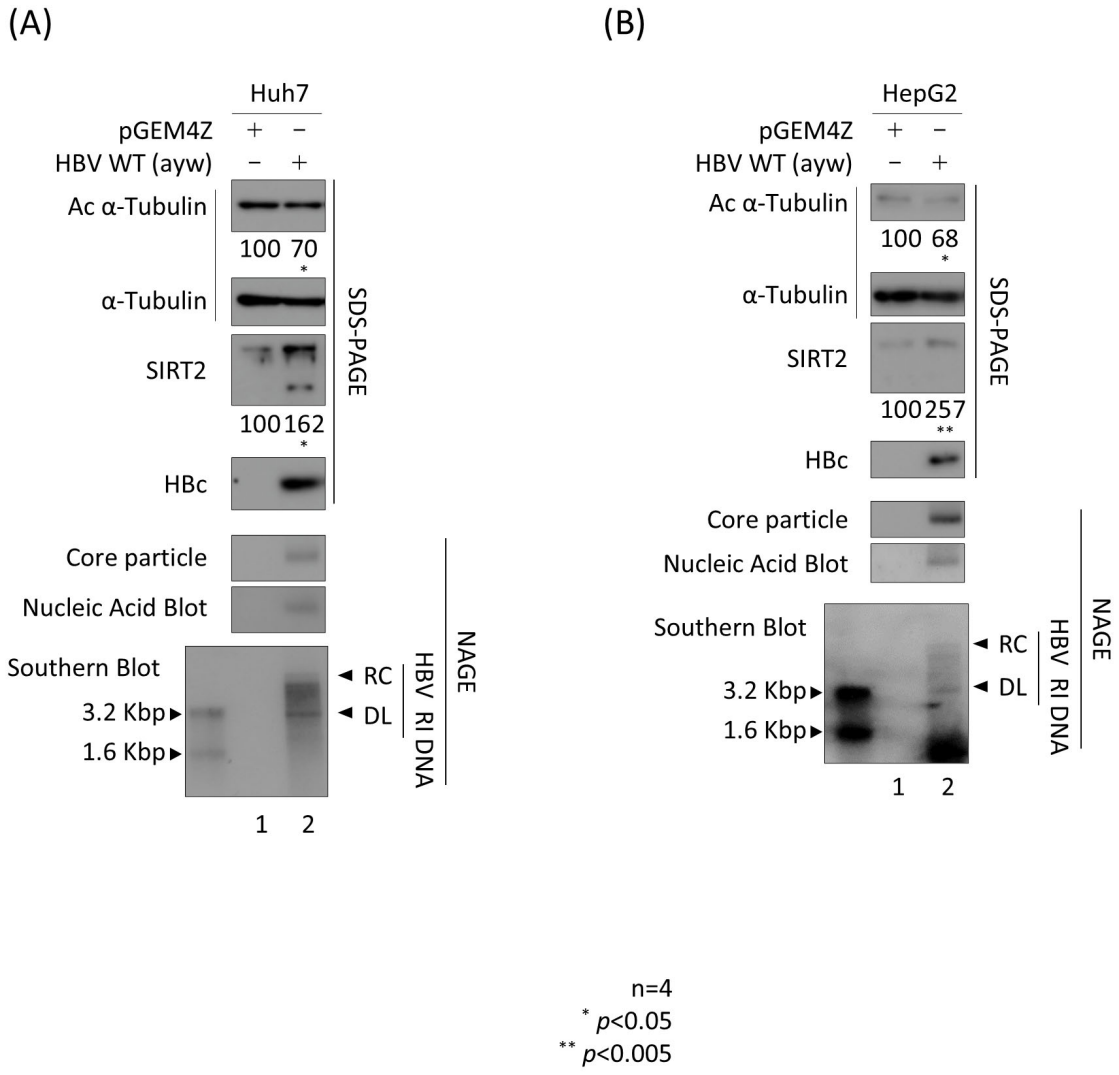
HBV replication increases endogenous SIRT2 levels, resulting in deacetylation of α-tubulin. **(A)** Huh7 or **(B)** HepG2 cells were transfected with 3 μg **(A)** or 8 μg **(B)** of 1.3mer HBV WT (lanes 2) and harvested at 72 h post-transfection. Lane 1 in A and B shows mock-transfected cells. Lysates were separated by SDS-PAGE and immunoblotted with appropriate primary antibodies (**Table 1**) to detect acetylated (Ac) α-tubulin, α-tubulin, endogenous SIRT2, and HBc (top to fourth panels). To detect core particles, lysates were subjected to 1% NAGE and then incubated with an anti-HBc antibody (fifth panels). *In situ* nucleic acid blotting was performed to detect HBV DNAs and RNAs inside the core particles (sixth panels). Southern blotting was performed to analyze HBV DNA synthesis (bottom panels). For *In situ* nucleic acid blotting, isolated core particles on PVDF membranes were treated with 0.2 N NaOH, hybridized with a random-primed ^32-^P-labeled full-length HBV specific probe, and subjected to autoradiography. For Southern blotting, HBV DNAs extracted from isolated core particles were separated, transferred to a nylon membrane, hybridized, and subjected to autoradiography. HBV replicative intermediate, partially double-stranded relaxed circular, and double-stranded linear DNAs are marked as HBV RI, RC, and DL, respectively. Relative expression of acetylated α-tubulin, endogenous cytoplasmic SIRT2, and HBc was quantified by the ImageJ 1.50b software program after normalizing to tubulin. Statistical significance was determined by Student’s t-test. ^*^, *p*<0.05; and ^**^, *p*<0.005 (relative to the control; n=4).

Previously, we reported that HBV replication increases expression of endogenous SIRT2, leading to deacetylation of α-tubulin (Piracha et al., 2018, 2020). To confirm the results of these previous reports (Piracha et al., 2018, 2020), we transfected Huh7 and HepG2 cells with the 1.3mer HBV wild-type (WT) (**Figures 1A, B**). HBV replicative intermediate (RI) DNA, including partially double-stranded relaxed circular relaxed circular (RC) and double-stranded linear (DL) DNA, were detected (**Figures 1A, B**, bottom panels, lanes 2). As expected, endogenous expression of SIRT2 was higher in HBV-transfected cells than in mock-transfected cells (Piracha et al., 2018, 2020) (**Figures 1A, B**; third panels, lane 1 *vs*. lane 2). Consistent with this, α-tubulin was deacetylated in HBV-transfected cells (Piracha et al., 2018, 2020) (**Figures 1A, B**, top panels, lane 1 *vs.* 2).

### SIRT2 inhibitor AGK2 inhibits replication of HBV

**Figure 2:**
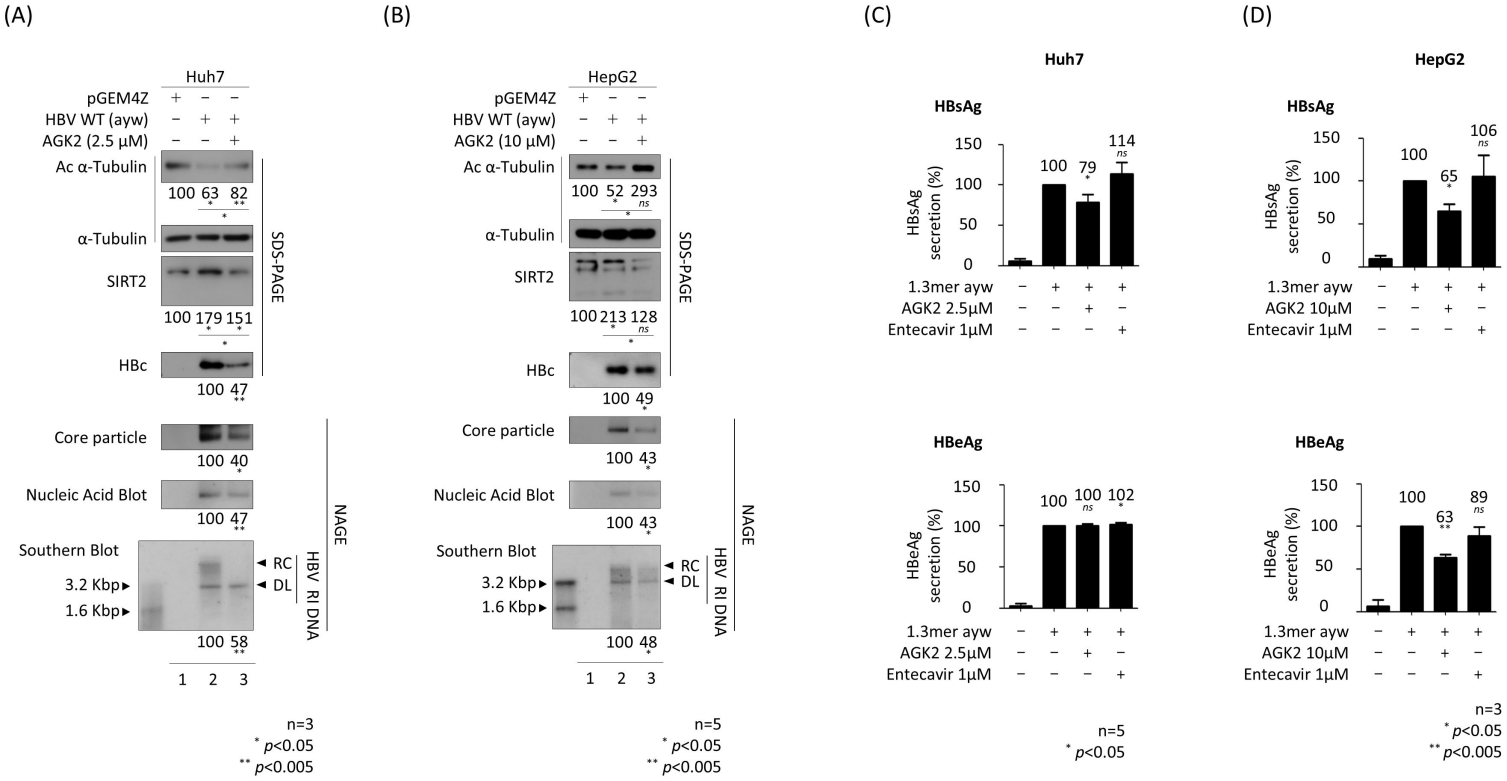
AGK2, a selective SIRT2 inhibitor, effectively suppresses HBV replication in HBV-transfected cells. **(A)** Huh7 cells were transfected with 3 μg of pGEM4Z (lane 1) or 1.3 mer HBV WT (ayw) (lanes 2 and 3). At the time of transfection, 2.5 μM of AGK2 (lane 3) was added for 72 h **(B)** HepG2 cells were transfected with 8 μg of pGEM4Z (lane 1) or 1.3 mer HBV WT (ayw) (lanes 2 and 3). At the time of transfection, 10 μM of AGK2 (lane 3) was added for 72 h Lysates were separated by SDS-PAGE and immunoblotted as described in **Figure 1**. Core particle analysis, *in situ* nucleic acid blotting, and Southern blotting were performed as described in **Figure 1**. **(C, D)** ELISAs measuring secretion of HBsAg and HBeAg into the culture supernatant of transfected-Huh7 and -HepG2 cells at 72 h post-transfection. Entecavir (1 μM) of was included at the time of transfection (as a control). Data derived from three **(A, D)** or five **(B, C)** independent experiments were analyzed using the ImageJ 1.50b software program. Statistical significance was determined using Student’s t-test. ^*^, *p*<0.05; and ^**^, *p*<0.005 (relative to the control).

Previously, we reported that AGK2, a selective SIRT2 inhibitor (Outeiro et al., 2007), inhibits HBV replication by downregulating AKT/GSK-3β/β-catenin signaling in HBV-transfected Huh7 and HepG2 cells (Piracha et al., 2018). This inhibitory activity of AGK2, particularly its effect on α-tubulin deacetylation, was validated by (Outeiro et al., 2007). Before initiating the current experiments, we performed MTT assays to evaluate AGK2 cytotoxicity in Huh7, HepG2, HepAD38, and HepG2.2.15 cells, confirming CC_50_ values above 100 µM for all cell lines (data not shown).This inhibitory activity of AGK2, particularly its effect on α-tubulin deacetylation, was validated by (Outeiro et al., 2007). Consistent with previous reports (Piracha et al., 2018; Yu et al., 2018), an MTT assay revealed that AGK2 was not cytotoxic at the concentrations used to treat Huh7 and HepG2 cells (**Supplementary Figure S1A**). However, during this study, slight morphological changes were observed in Huh7 cells treated with 10 µM AGK2; thus, we reduced the concentration to 2.5 µM for this cell line, which still effectively inhibited HBV replication. Next, we transfected the cells with 1.3mer HBV WT for 72 h and treated them simultaneously with AGK2. Consistent with the previous and above-mentioned results, we found that 1.3 mer HBV WT-transfected Huh7 and HepG2 cells harbored deacetylated α-tubulin and showed increased expression of SIRT2 (**Figures 2A, B**; top and third panels, lane 1 *vs.* 2); however, there was a significant reduction in SIRT2 expression upon treatment with AGK2 (**Figures 2A, B**; lane 2 *vs*. 3). Expression of HBc protein, core particle formation, and HBV DNA synthesis were reduced significantly by AGK2 treatment (**Figures 2A, B**; fourth to seventh panels, lane 2 *vs*. 3). A HBsAg ELISA conducted in Huh7 cells at 72 h post-transfection with HBV WT revealed that 2.5 μM AGK2 reduced HBsAg secretion significantly, while 1 μM entecavir did not (**Figure 2C**, upper panel). Unlike secretion of HBsAg, secretion of HBeAg secretion by Huh7 cells was not affected by AGK2 or entecavir (**Figure 2C**, lower panel). At 72 h post-transfection of HepG2 cells, the HBsAg and HBeAg ELISAs revealed that 10 μM AGK2 reduced HBsAg and HBeAg secretion significantly, whereas 1 μM entecavir did not (**Figure 2D**).

**Figure 3:**
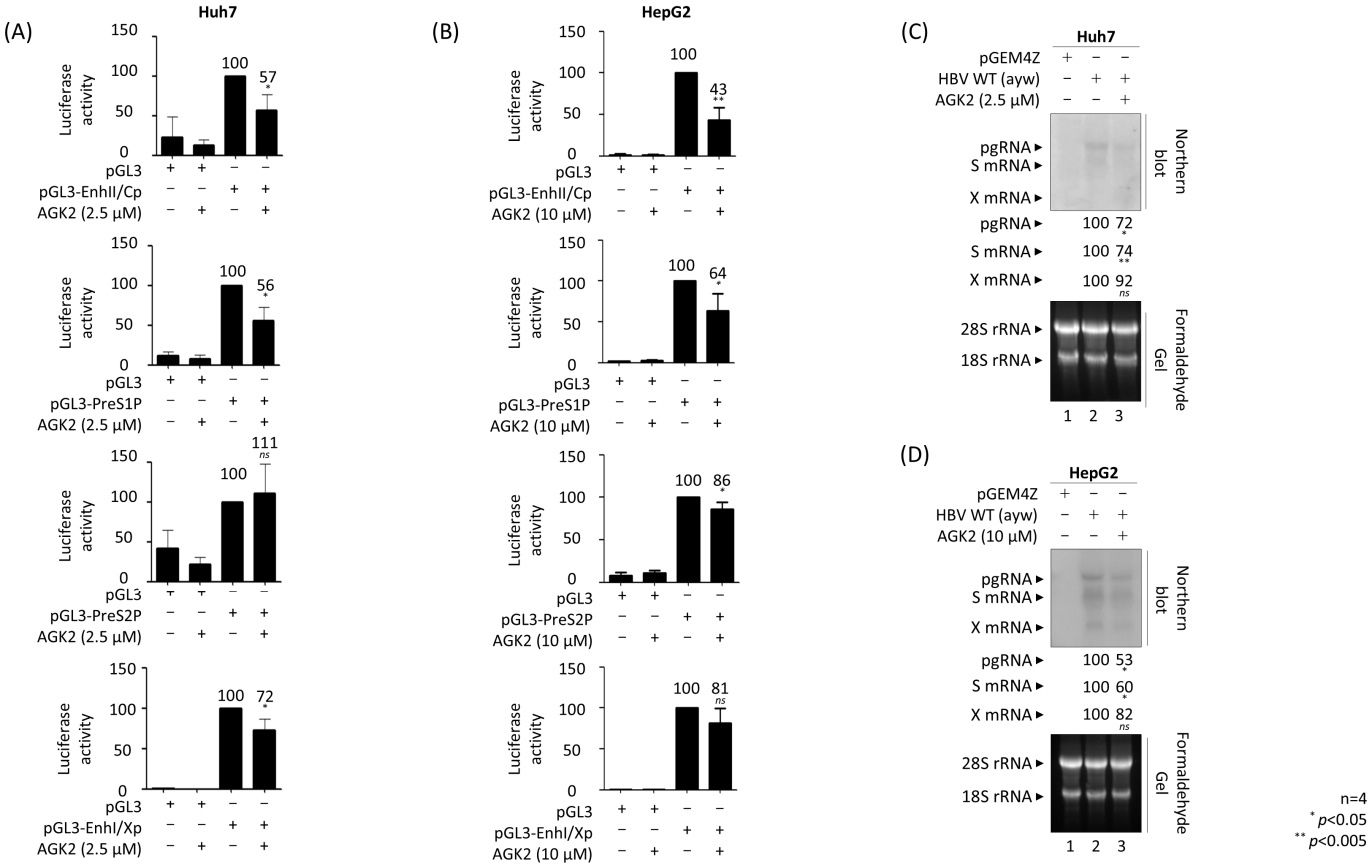
AGK2 effectively suppresses HBV transcriptional activity, thereby inhibiting production of HBV RNAs. **(A, B)** Luciferase reporter assays conducted after SIRT2 inhibition by AGK2 reveal reduced HBV enhancer and promoter activities. Huh7 **(A)** or HepG2 **(B)** cells were transiently transfected with 2 μg or 4 μg, respectively, of the specified luciferase reporter vectors. At the time of transfection, 2.5 μM of AGK2 was added to Huh7 cells **(A)**, and 10 μM of AGK2 was added to HepG2 cells **(B)**. Cell lysates were prepared and luciferase activity was measured at 72 h post-transfection. Luciferase activity was normalized to that of the respective control luciferase reporter vector. **(C, D)** Northern blotting shows the reduced levels of HBV transcripts upon inhibition of SIRT2 by AGK2. Huh7 **(C)** or HepG2 **(D)** cells were mock-transfected (lanes 1) or transiently transfected with 1.3 mer HBV WT (ayw) (lanes 2 and 3). At the time of transfection, 2.5 μM of AGK2 was added to Huh7 cells **(C)**, and 10 μM of AGK2 was added to HepG2 cells **(D)**. Total RNA was harvested at 72 h post-transfection, and northern blotting was conducted as described in the Methods. In brief, 20 µg of total RNAs were separated by 1% formaldehyde gel electrophoresis, transferred to a nylon membrane, hybridized, and subjected to autoradiography. The 3.5 kb pgRNA, 2.1 and 2.4 kb S mRNAs, and 0.7 kb X mRNA are indicated. Data from four **(A–D)** independent experiments were analyzed using the ImageJ 1.50b software program. Statistical significance was determined using Student’s t-test. ^*^, *p*<0.05; and ^**^, *p*<0.005 (relative to the control).

Since HBV DNA synthesis decreased in AGK2-treated HBV replicating cells (**Figures 2A, B**), we hypothesized that the transcriptional activity of HBV would also decrease. Therefore, we performed a luciferase reporter assay in AGK2-treated cells to examine HBV promoter activity (**Figures 3A, B**). The results showed that HBV transcriptional activity, including that of the enhancer II/core promoter (EnhII/Cp), the preS1 promoter, and the enhancer I/X promoter (EnhI/Xp), in Huh7 cells decreased upon treatment with AGK2 (**Figure 3A**). By contrast, there was no marked change in preS2 promoter activity following AGK2 treatment (**Figure 3A**). In AGK2-treated HepG2 cells, the activity of the EnhII/Cp, preS1, and preS2 promoters decreased (**Figure 3B**). The promoter activity of EnhI/Xp decreased, but the decrease was not statistically significant (**Figure 3B**). Consistent with results of the luciferase assays (**Figures 3A, B**), northern blotting revealed that the level of HBV pgRNA and subgenomic S mRNAs decreased in AGK2-treated, HBV-transfected Huh7 and HepG2 cells (**Figures 3C, D**, lane 2 *vs.* 3), suggesting that inhibition of SIRT2 by AGK2 represses HBV transcription, thereby decreasing HBV replication. The level of HBx mRNA also decreased, but not significantly (**Figures 3C, D**, lane 2 *vs*. 3).

### AGK2 reduces the amount of HBV cccDNA, thereby inhibiting HBV RNA and DNA syntheses and HBsAg and HBeAg secretion by HBV-infected HepG2-NTCP cells

**Figure 4:**
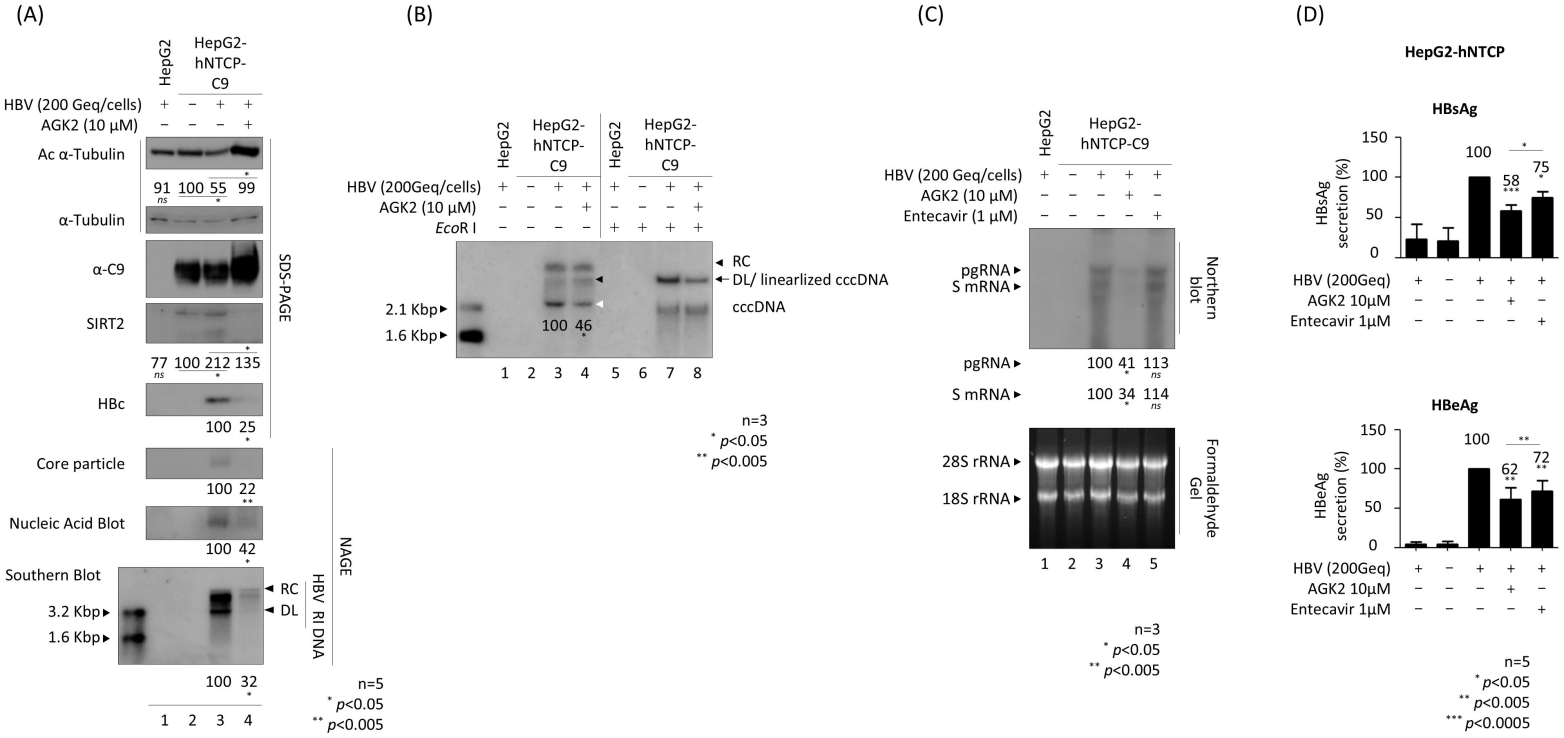
AGK2 suppresses cccDNA, leading to inhibition of RNA, DNA, HBsAg, and HBeAg by HBV-infected HepG2-hNTCP-C9 cells. **(A–C)** AGK2 suppresses HBV replication in the HBV infection system. HepG2 (lane 1) and HepG2-hNTCP-C9 (lanes 3 and 4) cells were infected with 2 × 10^2^ GEq of HBV per cell. Additionally, HepG2-hNTCP-C9 cells in lane 4 were treated with 10 μM AGK2. HepG2-hNTCP-C9 cells were mock-infected and incubated as described above (lane 2) **(A–C)**. All cells were incubated for 7 days. HBV-infected HepG2 cells (lane 1) and mock-infected HepG2-hNTCP-C9 cells (lane 2) served as negative controls. Relative expression of acetylated α-tubulin, endogenous SIRT2, HBc, core particles, and HBV DNA synthesis were analyzed as described in **Figure 1** using appropriate antibodies (**Table 1**). A mouse monoclonal anti-C9 antibody was used to detect hNTCP-C9 (**Table 1**). Relative expression was quantified using ImageJ 1.50b software after normalization to α-tubulin (loading control). **(B)** HBV cccDNA levels in HBV-infected cells decreased upon inhibition of SIRT2. Seven days after infection, cccDNA was extracted using a Hirt protein-free DNA extraction procedure, and cccDNA Southern blotting was performed. The white arrowhead indicates cccDNA, while the black arrow represents both the genome-length DL DNA and the linearized cccDNA generated by the *Eco*R I restriction enzyme. The black arrowheads indicate the DL DNA and RC DNA. **(C)** Northern blotting revealed that AGK2 reduces HBV RNA levels in HBV-infected cells. Total RNA was harvested at 5 days post-infection, and northern blotting was conducted as described in **Figures 3C, D** Entecavir (1 μM) was included at the time of infection (as a control; lane 5). **(D)** ELISAs were used to detect secreted HBsAg and HBeAg in culture supernatants at 5 days post-infection. Entecavir (1 μM) was included at the time of infection (as a control). Data from five **(A, D)** or three **(B, C)** independent experiments were analysis using the ImageJ 1.50b software program. Statistical significance was determined using Student’s t-test. ^*^, *p*<0.05; ^**^, *p*<0.005; and ^***^, *p*<0.0005 (relative to the control).

To investigate the effect of AGK2 in the HBV infection system, we established HepG2-hNTCP-C9 cells (**Figure 4A**, third panel, lanes 2–4). Briefly, HepG2‐hNTCP‐C9 stable cells were infected with 2 × 10^2^ GEq of HBV (**Figure 4A**, lanes 3–4) and treated with 10 μM AGK2 (lane 4), as described previously (Kim et al., 2022). Consistent with the above findings, and with previous reports (Piracha et al., 2018; Yu et al., 2018), we found that AGK2 was not cytotoxic in HepG2-NTCP‐C9 cells (**Supplementary Figure S1B**). In accordance with the transfection experiments (**Figures 1**, **2**), as well as previous infection experiments (Piracha et al., 2018, 2020), expression of endogenous SIRT2 was higher in HBV-infected cells than in non-infected cells (**Figure 4A**; fourth panel, lane 2 *vs*. 3). Correspondingly, α-tubulin was deacetylated in HBV-infected cells (**Figure 4A**; top panel, lane 2 *vs.* 3). Consistent with the results of the transfection experiments (**Figure 2**) (Piracha et al., 2018), AGK2 decreased endogenous SIRT2 levels significantly, and increased acetylation of α-tubulin (**Figure 4A**; top and fourth panels, lane 3 *vs.* 4). Expression of HBc protein, core particle formation, and HBV DNA synthesis were all reduced markedly by AGK2 treatment (**Figure 4A**; fifth to bottom panels, lane 3 *vs.* 4). Additionally, there was a significant reduction in the HBV cccDNA level in HBV-infected cells after AGK2 treatment (**Figure 4B**, lane 3 *vs.* 4, white arrowhead). Since AGK2 decreased HBV DNA synthesis and cccDNA levels in HBV-infected cells (**Figures 4A, B**), we next examined HBV RNA levels by northern blot analysis of AGK2-treated, HBV-infected HepG2-hNTCP-C9 cells (**Figure 4C**). The levels of HBV pgRNA and subgenomic S mRNAs fell significantly in AGK2-treated cells, but not in cells treated with 1µM entecavir (**Figure 4C**, lane 3 *vs*. lane 4 *vs*. lane 5).

In addition, HBsAg and HBeAg ELISAs revealed that secretion of HBsAg and HBeAg fell markedly after AGK2 treatment (**Figure 4D**). Unlike in transfected cells (**Figures 2C, D**), secretion of HBsAg and HBeAg by HBV-infected HepG2-hNTCP-C9 cells treated with 1 μM entecavir fell significantly, although to a much lesser extent than in AGK2-treated cells (**Figure 4D**). Collectively, these results indicate that AGK2 downregulates HBV cccDNA, leading to a decrease in syntheses of HBV RNAs and RI DNAs, and in secretion of HBsAg and HBeAg.

**Figure 5:**
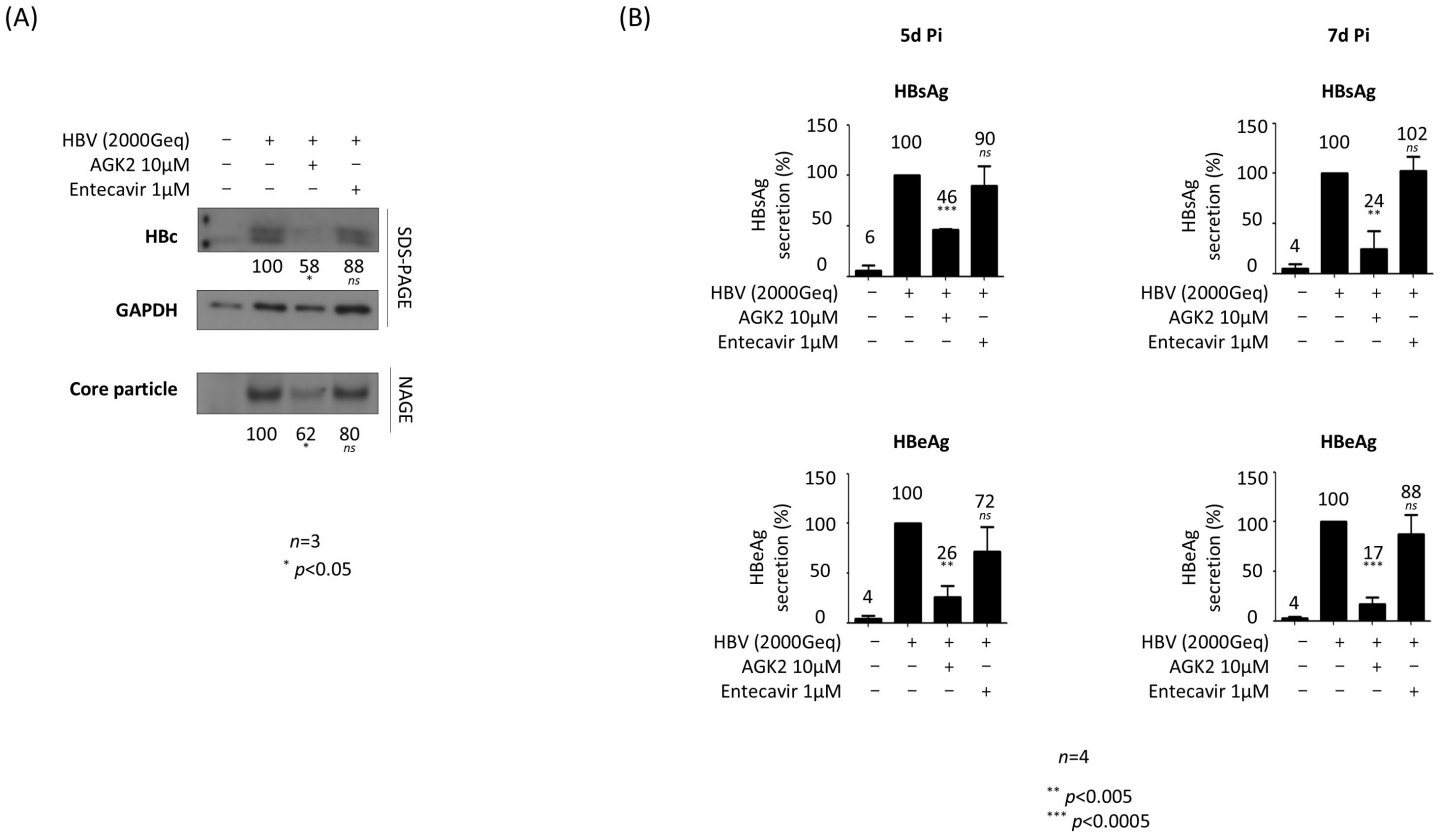
AGK2 suppresses HBV replication in HBV-infected PXB^®^ cells. **(A)** AGK2 suppresses HBc expression and core particle formation in HBV-infected PXB^®^ cells. PXB^®^ cells were infected with 2 × 10^3^ GEq of HBV per cell (lanes 2–4). **(A)** PXB^®^ cells were mock-infected and incubated as described in the Methods for PXB^®^ cells (lane 1). Additionally, at the time of infection PXB^®^ cells were treated with 10 μM of AGK2 (lane 3) or 1 μM of entecavir (lane 4; included as a control). **(B)** ELISAs were used to detect secretion of HBsAg and HBeAg into the culture supernatant at 5 days and 7 days post-infection. Data from five **(A)** or four **(B)** independent experiments were analysis using the ImageJ 1.50b software program. Statistical significance was determined using Student’s t-test. ^*^, *p*<0.05; ^**^, *p*<0.005; and ^***^, *p*<0.0005 (relative to the control).

We also infected PXB^®^ cells with HBV and then treated them with AGK2 (**Figure 5**). Briefly, PXB^®^ cells were infected with 2 × 10^3^ GEq of HBV (**Figure 5**), as described previously (Kim et al., 2022; Tang et al., 2024). As shown for AGK2-treated, HBV-infected HepG2‐hNTCP‐C9 cells, AGK2 treatment of infected PXB^®^ cells decreased HBc protein and core particle formation significantly (**Figure 5A**). Also, secretion of HBsAg and HBeAg fell markedly after AGK2 treatment; however, entecavir did not reduce HBsAg and HBeAg secretion significantly at 5 days or 7 days post-infection (**Figure 5B**).

### AGK2 increases recruitment of HKMTs and promotes deposition of their repressive epigenetic markers on cccDNA

**Figure 6:**
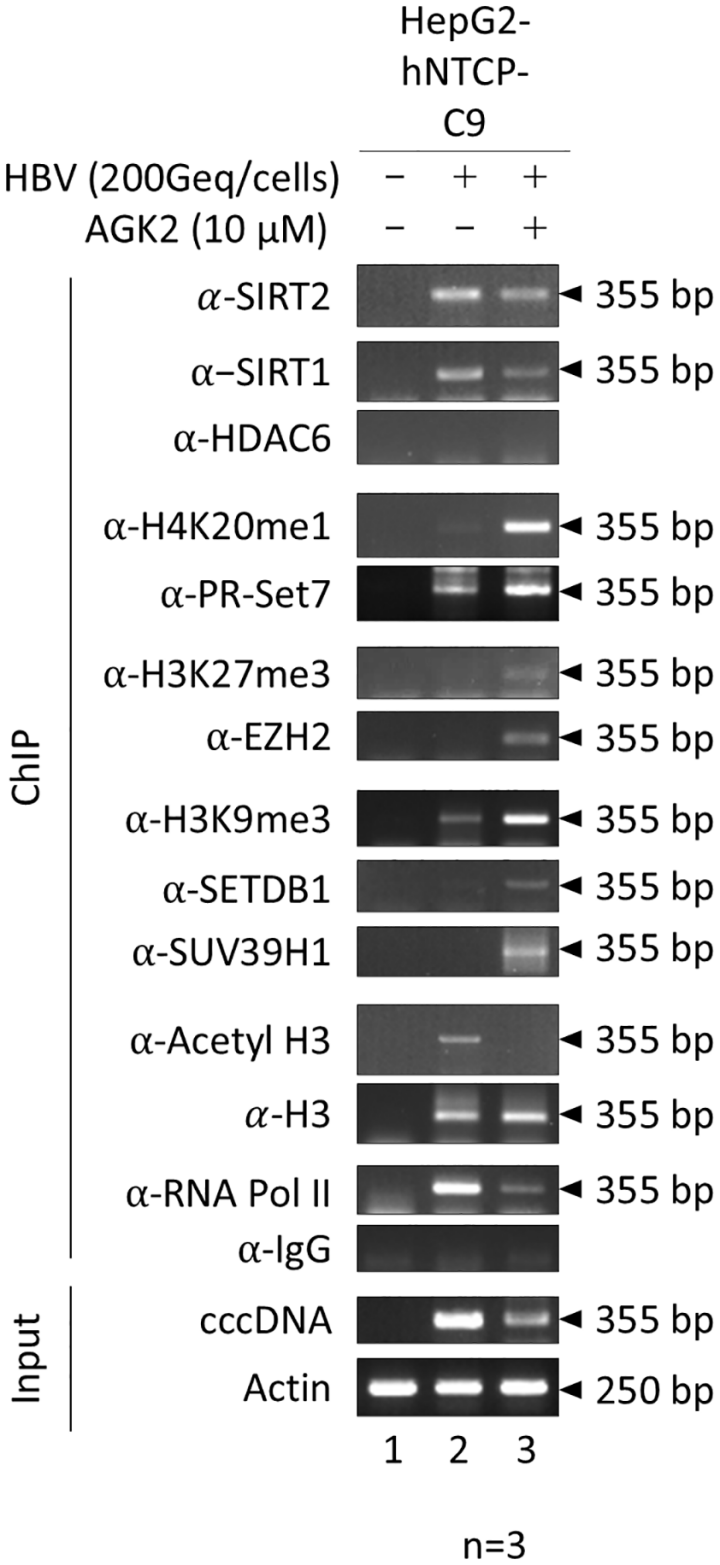
AGK2 increases recruitment of repressive HKMTs such as PR-Set7, EZH2, SETDB1, and SUV39H1 to HBV cccDNA, along with deposition of the respective epigenetic repressive markers. HBV cccDNA-recruited repressive HKMTs, and deposition of the respective epigenetic repressive markers, are increased by AGK2. HepG2-hNTCP-C9 cells were either mock-infected (lane 1) or infected with 2 × 10^2^ GEq of HBV (lanes 2 and 3), as described in **Figure 4**. Seven days after infection, chromatin solutions were prepared and subjected to immunoprecipitation using antibodies specific for SIRT2, SIRT1, HDAC6, H4K20me1, PR-Set7, H3K27me3, EZH2, H3K9me3, SETDB1, SUV39H1, H3, AcH3, or RNA Pol II (**Table 1**). Normal mouse or rabbit IgG antibodies were used as negative controls. Immunoprecipitated chromatin was analyzed by PCR. Actin levels were utilized to confirm equal loading of lysate samples. The data are representative of three independent experiments.

Next, we sought to explore the mechanism by which AGK2 inhibits viral transcription; in this context, we wanted to focus on mechanisms other than downregulation of the AKT/GSK-3β/β-catenin signaling pathway. Given that AGK2 suppresses transcriptional activity and reduces HBV RNA levels (**Figures 3**, **4C**), and the fact that overexpressed SIRT2.5 is recruited to cccDNA along with repressive HKMTs that deposit epigenetic repressive markers to suppress HBV transcription (Piracha et al., 2020), one possibility is that AGK2 inhibits viral transcription by altering the chromatin structure of cccDNA. To investigate this, we conducted a ChIP assay using HBV-infected HepG2-hNTCP-C9 cells to examine the association between AGK2 and cccDNA chromatin (**Figure 6**). Briefly, after infecting HepG2-hNTCP-C9 cells with 2 × 10^2^ GEq/cell of HBV and treating them with AGK2, we immunoprecipitated the chromatin using control IgG or specific antibodies. The immunoprecipitated pellet was subjected to semiquantitative PCR analysis (**Figure 6**) using selective cccDNA primers targeting the DR1 to DR2 region, initially designed by Werle-Lapostolle et al. (2004). These primers have subsequently been widely used for cccDNA PCR (Pollicino et al., 2006; Belloni et al., 2009, 2012), including our previously published work (Piracha et al., 2020). To begin with, constant amounts of an actin gene fragment (250 bp) were amplified (**Figure 6**, bottom), confirming consistent extraction of chromatin DNA from both mock- and HBV-infected cells. Amplification of input DNA revealed that cells treated with AGK2 had the lowest levels of cccDNA (**Figure 6**, fifteenth panel, lane 2 *vs.* 3), corroborating the findings shown in **Figure 4B**. AGK2-treated cells showed lower recruitment of SIRT2 to cccDNA; the levels were roughly comparable with the level of input DNA (**Figure 6**, first panel, lane 2 *vs*. 3). In accordance with a previous report (Belloni et al., 2012; Piracha et al., 2020), we found that SIRT1 was recruited to cccDNA at levels approximately comparable with those of input DNA (**Figure 6**, second panel). HDAC6, an exclusively cytoplasmic protein (Hubbert et al., 2002; Piracha et al., 2020) that cannot be recruited to cccDNA, was utilized as a negative control (**Figure 6**, third panel). Even though levels of endogenous cytoplasmic SIRT2 were reduced in AGK2-treated cells (**Figures 2A** and **4A**), recruitment of endogenous SIRT2 to the nucleus was approximately comparable with that of input cccDNA and SIRT1 (**Figure 6**, first and second panels, lane 2 *vs*. 3).

Post-translational modifications such as methylation and acetylation on H3 and H4 play a role in altering chromatin structure and regulating transcription of HBV cccDNA (Pollicino et al., 2006; Belloni et al., 2009; Levrero et al., 2009; Belloni et al., 2012). Heterochromatin-associated histone lysine modifications such as H4K20me1, H3K27me3, and H3K9me3 can induce chromatin condensation and transcriptional repression (Levrero et al., 2009; Piracha et al., 2020; Yang et al., 2020a; Andrisani, 2021). To assess whether AGK2 treatment correlates with a transcriptionally inactive cccDNA chromatin structure, we performed ChIP assays to determine deposition of repressive histone lysine methylations such as H4K20me1, H3K27me3, and H3K9me3 in AGK2-treated HBV-infected cells. Our data demonstrated that methylation of H4K20, H3K27, and H3K9 on the cccDNA minichromosome was higher in AGK2-treated cells than in control cells (**Figure 6**, fourth, sixth, and eighth panels, lane 2 *vs.* 3), indicating transcriptional repression upon AGK2 treatment. Overall, this finding demonstrates that AGK2 induces epigenetic modifications on cccDNA, as evidenced by increased levels of transcriptionally-repressive markers on cccDNA (**Figure 6**). Next, we investigated recruitment of repressive HKMTs, including PR-Set7, EZH2, SETDB1, and SUV39H1 (**Figure 6**, fifth, seventh, ninth and tenth panels, lane 2 *vs.* 3), all of which catalyze addition of a methyl group(s), resulting PR-Set7-mediated H4K20me1, EZH2-mediated H3K27me3, or SETDB1- and SUV39H1-mediated H3K9me3 (Beck et al., 2012; Piracha et al., 2020; Yang et al., 2022; Wang et al., 2023; Wu et al., 2023) in AGK2-treated HBV-infected cells. Consistent with methylation of H4K20, H3K27, and H3K9, AGK2 treatment increased recruitment of PR-Set7, EZH2, SETDB1, and SUV39H1 to cccDNA (**Figure 6**, fourth–tenth panels, lane 2 *vs.* 3).

Finally, we investigated the impact of AGK2 on recruitment of host RNA polymerase II, H3, and acetylated H3 (H3K9ac and H3K14ac) to cccDNA. We noticed a significant reduction in recruitment of RNA polymerase II to cccDNA upon AGK2 treatment (**Figure 6**, thirteenth panel, lane 2 *vs.* 3). The pattern for acetylated H3 was similar to that for RNA polymerase II (**Figure 6**, eleventh panels, lane 2 *vs*. 3), while recruitment of total H3 was not affected by AGK2 (**Figure 6**, twelfth panel, lane 2 *vs*. 3).

## Discussion

As yet, there is no cure for chronic hepatitis B, despite availability of many specific direct-acting antiviral drugs, including nucleos(t)ides analogs, capsid inhibitors, siRNAs, and gene editing agents (Dusheiko et al., 2023; Jeng et al., 2023).

**Figure 7:**
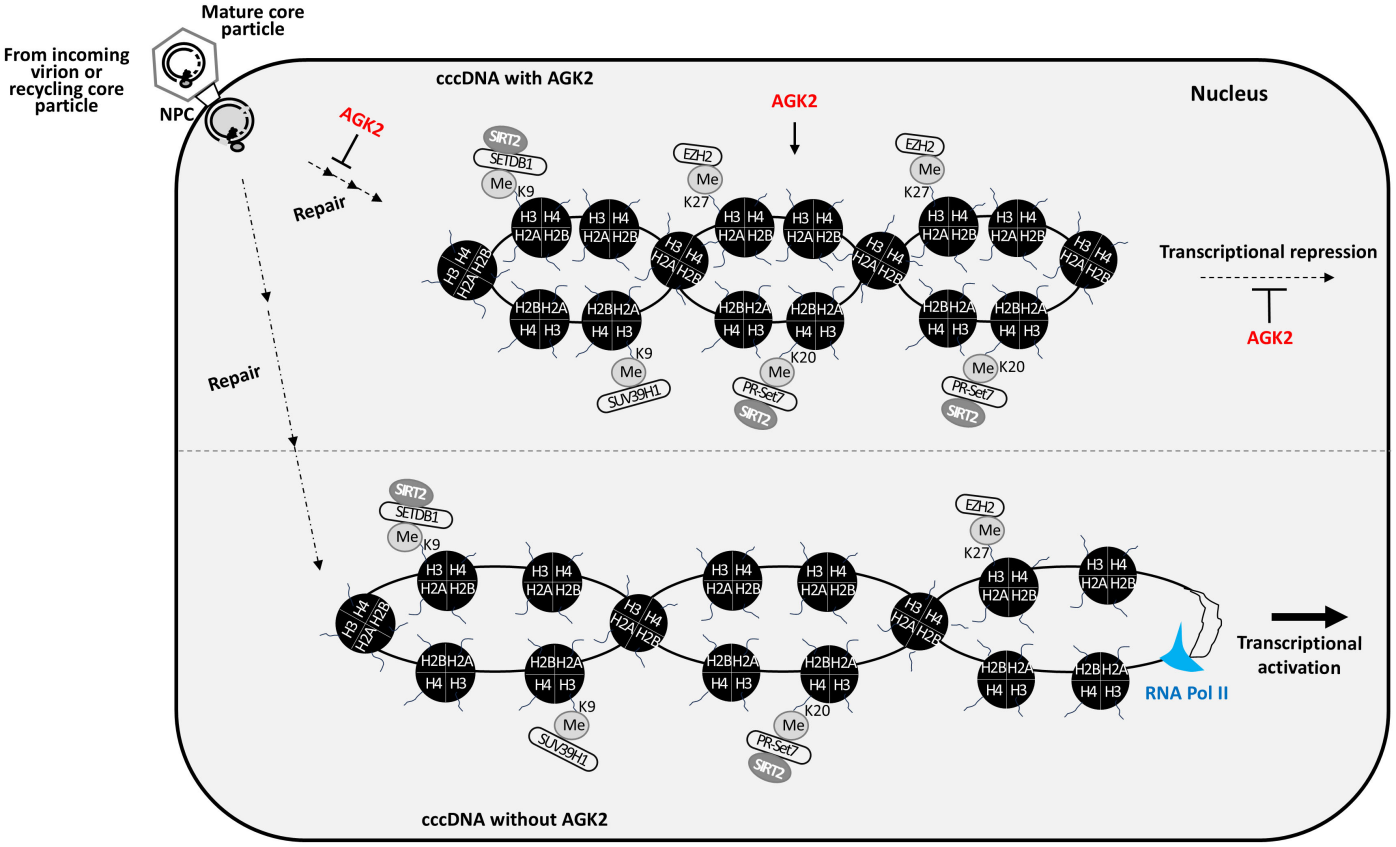
AGK2 suppresses HBV replication through multifaceted mechanisms. AGK2 affects the entire life cycle of HBV, initially by inhibiting cccDNA formation and then by inducing repressive epigenetic modification of cccDNA, which decreases RNA transcription and ultimately inhibits DNA synthesis. In the presence of AGK2, more repressive HKMTs such as SETDB1, SUV39H1, PR-Set7, and EZH2 are recruited onto cccDNA. SETDB1 and PR-Set7 interact with SIRT2 (Piracha et al., 2020). Also, AGK2 decreases recruitment of RNA polymerase II and acetylated H3 to cccDNA. As a consequence, SETDB1-mediated H3K9me3, SUV39H1-mediated H3K9me3, PR-Set7-mediated H4K20me1, and EZH2-mediated H3K27me3 cause repressive epigenetic modification of cccDNA, resulting in transcriptional repression.

The results presented herein show that the potent SIRT2 inhibitor AGK2 inhibits HBV replication through epigenetic modification of cccDNA chromatin (**Figures 5**-**7**) and cccDNA levels (**Figure 4B**). Consistent with a previous report (Piracha et al., 2018), we also demonstrate that AGK2-medited inhibition of HBV replication occurs not only through a decrease in HBc protein levels, core particle formation, HBV RNA transcription, and HBV DNA synthesis, but also via significant reduction in SIRT2 expression and a concomitant increase in α-tubulin acetylation (**Figures 2**-**4**). SIRT2 is primarily a cytoplasmic protein, although its localization can vary depending on the cellular context (Dryden et al., 2003; North and Verdin, 2007). Consistent with this, our previous results demonstrated that SIRT2.1 and SIRT2.5 play distinct roles in the epigenetic regulation of HBV-infected cells by modulating the presence of critical proteins involved in chromatin modification (Piracha et al., 2020). In brief, when we explored the differential impacts of overexpressing SIRT2.1 and SIRT2.5 on the epigenetic landscape of cccDNA, particularly on the marks H4K20me1, H3K9me3, and H3K27me3, our findings indicated that SIRT2.5 overexpression increases repressive epigenetic marks, whereas SIRT2.1 shows opposite results (Piracha et al., 2020). Based on these insights, we concluded that further silencing of SIRT2 was not really required for investigating changes in these particular epigenetic marks since we already demonstrated opposite epigenetic marks by SIRT2.1 and SIRT2.5. Consequently, by overexpression of SIRT2.1 and SIRT2.5, HBV RNA transcriptions were increased and decreased, respectively, indicating that these epigenetic marks are related to RNA transcriptions (Piracha et al., 2020).

A recent study reported a new allosteric SIRT2 inhibitor, FLS-359, which has broad spectrum antiviral activity against coronavirus, orthomyxovirus, flavivirus, hepadnavirus, and herpesvirus (Roche et al., 2023). When the anti-HBV activity of FLS-359 was investigated further, the data revealed that it inhibits cccDNA synthesis, cccDNA recycling, and HBV RNA transcription without significantly inhibiting RC DNA synthesis (Tang et al., 2024). Both AGK2 and FLS-359 inhibit HBsAg, unlike nucleos(t)ides analogs like entecavir (**Figures 2C, D**, **4D**, **5B**). Even though both AGK2 and FLS-359 inhibit cccDNA synthesis (**Figure 4B**) and HBV RNA transcription (**Figures 3**, **4C**) (Tang et al., 2024), FLS-359 (unlike AGK2) does not affect SIRT2 levels or α-tubulin acetylation (Tang et al., 2024) (**Figures 2A, B**, **4**). Although both AGK2 and FLS-359 inhibit HBV RNA transcription (**Figures 3**, **4C**) (Tang et al., 2024), FLS-359 does not affect RC DNA synthesis significantly (Tang et al., 2024); rather, AGK2 reduces RC DNA levels significantly (**Figures 2A, B**, **4A**).

Tang et al (Tang et al., 2024), not only compared the anti-HBV activity of AGK2 (in terms of HBsAg and HBeAg secretion), but also that of other SIRT2 inhibitors such as thiomyristoyl, cambinol, and SirReal2; the authors demonstrated that all of these inhibitors decreased secretion of HBsAg and HBeAg. Cambinol is an inhibitor of both SIRT1 and SIRT2 (Giordano et al., 2023), although its mode of action against HBV might be different from that of AGK2, thiomyristoyl, and SirReal2. Our unpublished results suggest that the mechanisms underlying the anti-HBV effect of AGK2, thiomyristoyl, or SirReal2 might be different, since thiomyristoyl does not inhibit HBV RNA synthesis (unpublished). Our data, and those of Tang et al (Tang et al., 2024), indicate that SIRT2 is a potential therapeutic target for combating HBV infection.

Although SIRT2 is known to mediate histone deacetylation (Vaquero et al., 2006; Das et al., 2009; Eskandarian et al., 2013), the inhibition of SIRT2 by AGK2 has been specifically validated only in the context of α-tubulin deacetylation (Outeiro et al., 2007) (**Figures 2A, B**, **4A**, top panels). Thus, it remains unclear whether AGK2 directly affects SIRT2-mediated histone acetylation. Our rationale is based on the observation that AGK2 inhibits SIRT2 (**Figures 2A, B**, **4A**, third panels), leading to a suppression of HBV transcription (**Figures 3**, **4C**) which coincides with increased deposition of repressive epigenetic markers, reduced histone H3 acetylation, and decreased RNA polymerase II recruitment to cccDNA (**Figure 6**).

Most importantly, we found that AGK2 has a significant impact on the chromatin structure of cccDNA, as evidenced by the ChIP assays. In AGK2-treated cells, increased deposition of repressive histone lysine methylations (H4K20me1, H3K9me3, and H3K27me3) on cccDNA led to transcriptional repression. Additionally, increased recruitment of relevant HKMTs (PR-Set7, EZH2, SETDB1, and SUV39H1) to cccDNA further confirmed transcriptional repression, resulting in transcriptionally silenced cccDNA. We speculate that reduced RC DNA synthesis mediated by AGK2 may be due to reduced HBV RNA transcription (**Figures 2A, B**, **3**, **4B**, **7**) caused by repressive epigenetic changes on cccDNA chromatin by PR-Set7-mediated H4K20me1, EZH2-mediated H3K27me3, or SETDB1- and SUV39H1-mediated H3K9me3 (**Figures 6**, **7**). These repressive epigenetic changes on cccDNA chromatin are unprecedented. Our results suggest that AGK2 might be a potential epidrug that induces a heterochromatin state in cccDNA to achieve a functional cure of CHB. Thus, AGK2 acts by inducing repressive epigenetic changes, as well as through the AKT/GSK-3β/β-catenin signaling pathway (Piracha et al., 2018).

## Conclusions

Our findings highlight AGK2 as a potent inhibitor of HBV replication, which acts through epigenetic repression of cccDNA chromatin structure, by inhibiting viral transcription, and by reducing cccDNA levels. The study provides valuable insights into the potential use of SIRT2 inhibitors as therapeutic agents against HBV infection.

## Methods

### Plasmids

The HBV wild type (WT) 1.3 mer (subtype ayw) plasmid was kindly provided by Dr. Ryu WS (Yonsei Univ, South Korea). The pcDNA6.1-human NTCP (hNTCP)-C9 plasmid was a kind gift from Dr. W. Li (National Institute of Biological Sciences, China) (Yan et al., 2012). The pCDH-hNTCP-C9 plasmid was generated from pcDNA6.1-hNTCP-C9 (Piracha et al., 2018). The luciferase reporter plasmids pGL3‐EnhII/Cp, pGL3‐EnhI/Xp, pGL3‐ preS1p, and pGL3‐preS2p were described previously (Saeed et al., 2019).

### Cell culture and transfection with plasmids

HepG2, HepG2-hNTCP-C9, HepAD38, Huh7, and HEK293T cells were cultured in Dulbecco’s modified Eagle’s medium (DMEM; Gibco) supplemented with 10% (v/v) heat-inactivated fetal bovine serum (FBS; Gibco-BRL) and 1% (v/v) penicillin‐streptomycin (Gibco). The cells were maintained in a humidified atmosphere at 37°C/5% CO_2_. HepAD38 cells were kindly provided by Christopher Seeger (Fox Chase Cancer Center) and cultured in the same medium as HepG2 cells, but with addition of 1 mg/ml G418 (G0175.0005, Duchefa Biochemie) and 2 µg/ml tetracycline (T7660-5G, Sigma-Aldrich). The tetracycline was removed to induce HBV transcription and production of virions for infection (Ladner et al., 1997). Primary human hepatocytes (PXB^®^) cells were maintained in modified dHCGM medium (Yamasaki et al., 2006) (PhoenixBio USA Corporation). For transfection, Huh7 (5 × 10^5^) cells cultured in 6 cm dishes were incubated with a mixture comprising 3 μg of the 1.3mer HBV WT (ayw) or a control plasmid, 9 μg of polyethylenimine (PEI, Polysciences, 23966‐2), and 200 μl of Opti-MEM (Gibco, 31985062). In addition, HepG2 (2 × 10^6^) cells cultured in 6 cm dishes were transfected with a mixture containing 8 μg of the 1.3mer HBV WT (ayw) or a control plasmid, 40 μg of PEI, and 200 μl of Opti-MEM. Cells were inoculated with the transfection mixture at 24 h post-seeding. The mixture was replaced with fresh medium at 24 h post-transfection. At 72 h post-transfection, cells were lyzed in 0.2% NP‐40 (IGEPAL)‐TNE (10 mM Tris‐HCl [pH 8.0], 50 mM NaCl, 1 mM EDTA) buffer, as described previously (Kim et al., 2022).

### Preparation of HBV, and infection of cells

The HBV virions used for infection were harvested from HepAD38 cells as described, with minor modifications (Kim et al., 2022). Stable HepG2‐hNTCP‐C9 cells were established using a lentiviral expression system as described previously (Kwon et al., 2023). Briefly, 1 × 10^6^ (in 6 cm collagen-coated (Corning, 354249) plates) or 3 × 10^6^ (in 10 cm collagen-coated plates) HepG2 or HepG2‐hNTCP‐C9 cells, respectively, were infected with 200 genome equivalents (GEq) of HBV virions per cell in medium containing 4% polyethylene glycol (PEG, Affymetrix, 25322- 68-3) and 2.5% dimethyl sulfoxide (DMSO, Sigma Aldrich, D8418). PXB^®^ (4 × 10^5^ cells/well in a 24 well-plate) cells were infected with 2,000 GEq of HBV. After 24 h, the cells were washed thoroughly (at least three times) with phosphate-buffered saline (PBS) and maintained in the same medium containing 2.5% DMSO. The medium was refreshed every second day.

### Treatment with AGK2

To examine the effects of AGK2 (catalog number A8231; Sigma-Aldrich) (Outeiro et al., 2007) on HBV replication, the compound was dissolved in DMSO (4mM AGK2 stock solution) and used to treat transfected/HBV-infected cells for 72 h, or for 5 or 7 days. In brief, Huh7, HepG2, or HepG2‐hNTCP‐C9 or PXB^®^ cells were either transiently transfected or infected as described above. AKG2 (at the indicated concentrations) was added at the time of transfection or infection for the indicated times, followed by preparation of cell lysates as described (Kim et al., 2022). The cytotoxic effect of AGK2 were assessed in an MTT (3‐[4,5‐dimethylthiazol‐2‐yl]‐2,5‐diphenyltetrazolium bromide) assay, as described previously (Jung et al., 2015), or in an MTS assay according to the manufacturer’s instructions (Cell Proliferation; Colorimetric; ab197010).

### Extraction of HBV cccDNA

To investigate the effects of AGK2 on formation of HBV cccDNA, HBV cccDNA was extracted from cells using the Hirt protein‐free DNA extraction procedure, as previously described (Cai et al., 2013), but with minor modifications (Kim et al., 2022). Briefly, 3 × 10^6^ HepG2 or HepG2‐hNTCP‐C9 cells, cultured on collagen‐coated 10 cm dishes, were infected with HBV and treated with 10 μM AGK2 for 7 days. Cell lysates were prepared as described (Cai et al., 2013; Kim et al., 2022), and cccDNA was precipitated using ethanol and then analyzed by Southern blotting. To further validate the authenticity of HBV cccDNA, the Hirt DNA sample was heated to 85°C for 5 min. The HBV DNA extracted from the Hirt protein-free DNA extraction sample includes cccDNA (2.1 Kbp), DL DNA (3.2 Kbp), and protein-free RC DNA (above 3.2 Kbp). Heating the Hirt DNA sample denatures the RC and DL DNAs, leaving cccDNA unaffected. Consequently, the electrophoretic mobility of the cccDNA does not change. In addition, the heat-treated DNA sample was digested with *Eco*R I to linearize the cccDNA, resulting in a genome-length double-stranded DNA of 3.2 Kbp.

### SDS-PAGE, immunoblotting, and enzyme-linked immunosorbent assays

The protein content of the cell lysates prepared in 0.2% NP‐40‐TNE buffer was measured using a Bradford assay (Kim et al., 2022). Lysates containing equal quantities of protein were separated by sodium dodecyl sulfate polyacrylamide gel electrophoresis (SDS‐PAGE) in 10% or 12% gels and transferred to PVDF membranes (Millipore). The membranes were blocked with 4% skim milk, incubated with appropriate primary antibodies, and then incubated with secondary horseradish peroxidase (HRP)‐conjugated goat anti-rabbit or HRP‐conjugated anti-mouse antibodies (**Table 1**). Proteins were visualized by enhanced chemiluminescence (ECL, GE Healthcare Life Sciences, RPN2106). Relative band intensities were quantified using ImageJ version 1.50b. Culture supernatants from 1.3 mer HBV WT-transfected Huh7 or HepG2 cells, and from HBV infected-HepG2‐hNTCP‐C9 or PXB^®^ cells, were collected at the indicated times and used in enzyme-linked immunosorbent assays (ELISA) designed to detect HBsAg (WANTAI HBsAg ELISA kit, WB-2296) and HBeAg (WANTAI HBeAg ELISA kit, WB-2496).

**Table 1:** Antibodies used in this study.

Antibody Target	Species	Experiment	Dilution	Supplier	Catalog# (Ref)
HBc	Rabbit polyclonal	SDS-PAGE-IB/NAGE-IB	1:1,000	In-house	(49)
α-Tubulin	Mouse monoclonal	SDS-PAGE-IB	1:1,000	Merck Millipore,	05-829
Acetylated α-tubulin	Mouse monoclonal	SDS-PAGE-IB	1:1,000	Sigma-Aldrich	T6793
Rhodopsin-C9	Mouse monoclonal	SDS-PAGE-IB	1:1,000	Millipore	MAB5356
horseradish peroxidase (HRP)‐conjugated anti-rabbit	Goat polyclonal	SDS-PAGE-IB/NAGE-IB	1:5,000	Thermo Fisher Scientific	31460
HRP‐conjugated anti-mouse	Goat polyclonal	SDS-PAGE-IB	1:5,000	KPL	474‐1802
SIRT2	Rabbit polyclonal	SDS-PAGE-IB/ChIP	1:1,000/1:100	Santa Cruz	sc-20966
SIRT1	Mouse monoclonal	ChIP	1:100	Santa Cruz	sc-74465
HDAC6	Rabbit polyclonal	ChIP	1:100	Santa Cruz	sc-11420
H3K27me3	Mouse monoclonal	ChIP	1:100	Cell Signaling Technology	9733
H3K9me3	Rabbit polyclonal	ChIP	1:100	Abcam	ab8898
H4K20me1	Rabbit monoclonal	ChIP	1:100	Abcam	ab177188
RNA Pol II	Mouse monoclonal	ChIP	1:100	Abcam	ab817
SUV39H1	Rabbit monoclonal	ChIP	1:100	Cell Signaling Technology	8729
PR-Set7	Rabbit polyclonal	ChIP	1:100	Abcam	ab230683
EZH2	Rabbit monoclonal	ChIP	1:100	Abcam	ab191250
SETDB1	Rabbit polyclonal	ChIP	1:100	Abcam	ab12317

### Native agarose gel electrophoresis and immunoblotting

To visualize core particles, cell lysates prepared in 0.2% NP‐40‐TNE buffer were separated by 1% native agarose gel electrophoresis (NAGE), transferred to a PVDF membrane, and immunoblotted with a rabbit polyclonal anti-HBc antibody (Jung et al., 2012) followed by an HRP-conjugated anti-rabbit secondary antibody. The core particles were visualized using ECL, and relative band intensities were quantified using ImageJ version 1.50b.

### Northern, Southern, and *in situ* nucleic acid blotting

Total RNA was extracted from Huh7, HepG2, or HepG2-hNTCP-C9 cells using Trizol (Ambion, 15596018) at 3 days post-transfection or at 5 days post-infection. Next, 20 μg of total RNA was denatured at 65°C for 10 min and separated in 1% agarose gels (Invitrogen, 16500100) containing 18% formaldehyde (Sigma Aldrich, F8775) and 1 × morpholinopropanesulfonic acid (MOPS) buffer (200 mM MOPS, 10 mM EDTA, 50 mM sodium acetate [pH 7.0]). The RNA was transferred to nylon membranes (Roche, 11417240001) and hybridized at 68°C for 4 h with a random‐primed ^32^P‐labeled probe specific for full‐length HBV sequences.

HBV DNA was extracted from isolated core particles at 3 days post-transfection or 7 days post-infection, electrophoresed on 1% NAGE gels, transferred to nylon membranes, and subjected to hybridization as described above. Next, *in situ* nucleic acid blotting was performed to analyze HBV nucleic acids (including pgRNA and RI DNAs) inside core particles. The PVDF membrane used for immunoblotting of core particles was treated for 60 s with 0.2 N NaOH, washed quickly with distilled water, UV-crosslinked (XL-1500 UV CROSSLINKER, Spectrolinker™), and subjected to hybridization as described above. Relative band intensities were quantified using ImageJ version 1.50b.

### Luciferase assay

To determine the effects of AGK2 on HBV transcription, Huh7 (2.5 × 10^5^) or HepG2 (1 × 10^6^) cells were seeded onto 6-well plates and transfected with 2 μg or 4 μg the luciferase report vectors pGL3-null, pGL3-EnhII/Cp, pGL3-PreS1p or pGL3-PreS2p, and pGL3-EnhI/Xp. The cells were treated with AGK2 at the time of transfection. After 72 h, cells were lysed using 1 × luciferase cell culture lysis reagent (Promega, E153A) and luciferase activity measured by adding luciferin (Promega). Plates were read in a luminometer (Molecular Devices, EPOCH2NS).

### Chromatin immunoprecipitation of HBV cccDNA

ChIP of HBV cccDNA from cells at 7 days post-infection was conducted as described previously, with minor modifications (Piracha et al., 2020; Saeed et al., 2021). Briefly, 3 × 10^6^ HepG2‐hNTCP‐C9 cells were seeded on 10 cm collagen‐coated dishes, infected with HBV, and treated with 10 μM AGK2 as described above. At 7 days post-infection, nuclear pellets derived from cell lysates (0.2% NP‐40‐TNE) were fixed for 30 min at 4°C with 1 ml of 1% formaldehyde-containing buffer (20 mM Tris-HCl [pH 8.0], 20 mM KCl, 3 mM MgCl_2_, 1 mM PMSF, 1 mM DTT). The fixed pellet was then subjected to immunoprecipitation overnight at 4°C with 1 µg of the indicated specific antibody (**Table 1**) or with normal mouse or rabbit IgG (negative controls), as described previously (Pollicino et al., 2006; Piracha et al., 2020; Saeed et al., 2021). The immunoprecipitated protein-DNA complexes were eluted (1% SDS, 0.1 M NaHCO_3_) and reverse cross-linked at 60°C for 4 h. The immunoprecipitated DNA was purified by treatment with proteinase K (Sigma Aldrich, P2308), followed by phenol-chloroform extraction and ethanol precipitation. The purified DNA was then dissolved in nuclease-free water (Invitrogen, AM9932). Input samples were prepared from sonicated chromatin solutions. Following measurement of the optical density at 260 nm (OD260), the DNA concentration was adjusted to 50 ng. Actin was utilized to facilitate equal loading of protein from lysates. The forward and reverse primers used to target actin gene were 5’-CAT GTA CGT TGC TAT CCA GGC-3’ and 5’-CTC CTT AAT GTC ACG CAC GAT-3’, respectively. The forward and reverse primers targeting cccDNA were 5’-CTC CCC GTC TGT GCC TTC T-3’ and 5’-GCC CCA AAG CCA CCC AAG-3’, respectively. Samples were subjected to both PCR (GeneAmp PCR system 2700; Applied Biosystems, Thermo Fisher Scientific).

### Statistical analysis

Data are presented as the mean ± SD from a minimum of three independent experiments. Statistical comparisons of mean values were performed using Student’s t-test. The luciferase reporter assay data were analyzed using GraphPad Prism version 9.0. P values of 0.05 were considered statistically significant.

## Data Availability

The original contributions presented in the study are included in the article/**Supplementary Material**. Further inquiries can be directed to the corresponding author.

## Supplementary material

Supplementary Figure 1 AGK2 is not cytotoxic to Huh7, HepG2, HepG2-NTCP-C9, or PXB^®^ cells. **(A)** At 24 h after seeding, Huh7 and HepG2 cells were treated with AGK2 for 72 h. **(B, C)** At 24 h after seeding, HepG2-NTCP-C9 and PXB^®^ cells were treated with AGK2 for 7 days. MTT assay **(A)** or MTS **(B, C)** assays were performed to measure cell viability. Data from three **(A, B)** or five **(C)** independent experiments were analyzed using the ImageJ 1.50b software program. The bars represent the mean ± SD of three **(A, B)** or five **(C)** independent experiments. Statistical significance was determined using Student’s t-test. *ns*, not significant relative to the control.

## References

[B1] Alqarni M. H. Foudah A. I. Muharram M. M. Labrou N. E. (2021). The pleiotropic function of human sirtuins as modulators of metabolic pathways and viral infections. Cells 10, 460. doi: 10.3390/cells10020460 33669990 PMC7927137

[B2] Andrisani O. (2021). Epigenetic mechanisms in hepatitis B virus-associated hepatocellular carcinoma. Hepatoma Res. 7, 12. doi: 10.20517/2394-5079.2020.83 33614973 PMC7894648

[B3] Beck D. B. Oda H. Shen S. S. Reinberg D. (2012). PR-Set7 and H4K20me1: at the crossroads of genome integrity, cell cycle, chromosome condensation, and transcription. Genes Dev. 26, 325–337. doi: 10.1101/gad.177444.111 22345514 PMC3289880

[B4] Belloni L. Allweiss L. Guerrieri F. Pediconi N. Volz T. Pollicino T.. (2012). IFN-α inhibits HBV transcription and replication in cell culture and in humanized mice by targeting the epigenetic regulation of the nuclear cccDNA minichromosome. J. Clin. Invest. 122, 529–537. doi: 10.1172/jci58847 22251702 PMC3266786

[B5] Belloni L. Pollicino T. De Nicola F. Guerrieri F. Raffa G. Fanciulli M.. (2009). Nuclear HBx binds the HBV minichromosome and modifies the epigenetic regulation of cccDNA function. Proc. Natl. Acad. Sci. U.S.A. 106, 19975–19979. doi: 10.1073/pnas.0908365106 19906987 PMC2775998

[B6] Budayeva H. G. Rowland E. A. Cristea I. M. (2016). Intricate roles of mammalian sirtuins in defense against viral pathogens. J. Virol. 90, 5–8. doi: 10.1128/jvi.03220-14 26491165 PMC4702534

[B7] Cai D. Nie H. Yan R. Guo J. T. Block T. M. Guo H. (2013). A southern blot assay for detection of hepatitis B virus covalently closed circular DNA from cell cultures. Methods Mol. Biol. 1030, 151–161. doi: 10.1007/978-1-62703-484-5_13 23821267 PMC5060941

[B8] Carafa V. Rotili D. Forgione M. Cuomo F. Serretiello E. Hailu G. S.. (2016). Sirtuin functions and modulation: from chemistry to the clinic. Clin. Epigenet. 8, 61. doi: 10.1186/s13148-016-0224-3 PMC4879741 27226812

[B9] Dandri M. (2020). Epigenetic modulation in chronic hepatitis B virus infection. Semin. Immunopathol. 42, 173–185. doi: 10.1007/s00281-020-00780-6 32185454 PMC7174266

[B10] Das C. Lucia M. S. Hansen K. C. Tyler J. K. (2009). CBP/p300-mediated acetylation of histone H3 on lysine 56. Nature 459, 113–117. doi: 10.1038/nature07861 19270680 PMC2756583

[B11] Dryden S. C. Nahhas F. A. Nowak J. E. Goustin A. S. Tainsky M. A. (2003). Role for human SIRT2 NAD-dependent deacetylase activity in control of mitotic exit in the cell cycle. Mol. Cell Biol. 23, 3173–3185. doi: 10.1128/mcb.23.9.3173-3185.2003 12697818 PMC153197

[B12] Dusheiko G. Agarwal K. Maini M. K. (2023). New approaches to chronic hepatitis B. N Engl. J. Med. 388, 55–69. doi: 10.1056/NEJMra2211764 36599063

[B13] Eskandarian H. A. Impens F. Nahori M. A. Soubigou G. Coppée J. Y. Cossart P.. (2013). A role for SIRT2-dependent histone H3K18 deacetylation in bacterial infection. Science 341, 1238858. doi: 10.1126/science.1238858 23908241

[B14] Giordano D. Scafuri B. De Masi L. Capasso L. Maresca V. Altucci L.. (2023). Sirtuin inhibitor cambinol induces cell differentiation and differently interferes with SIRT1 and 2 at the substrate binding site. Biomedicines 11, 1624. doi: 10.3390/biomedicines11061624 37371719 PMC10295654

[B15] Hackett B. A. Dittmar M. Segrist E. Pittenger N. To J. Griesman T.. (2019). Sirtuin inhibitors are broadly antiviral against arboviruses. mBio 10, e01446-19. doi: 10.1128/mBio.01446-19 31289184 PMC6747726

[B16] Hubbert C. Guardiola A. Shao R. Kawaguchi Y. Ito A. Nixon A.. (2002). HDAC6 is a microtubule-associated deacetylase. Nature 417, 455–458. doi: 10.1038/417455a 12024216

[B17] Jeng W. J. Papatheodoridis G. V. Lok A. S. F. (2023). Hepatitis B. Lancet 401, 1039–1052. doi: 10.1016/s0140-6736(22)01468-4 36774930

[B18] Jiang H. Cheng S. T. Ren J. H. Ren F. Yu H. B. Wang Q.. (2019). SIRT6 inhibitor, OSS_128167 restricts hepatitis B virus transcription and replication through targeting transcription factor peroxisome proliferator-activated receptors α. Front. Pharmacol. 10. doi: 10.3389/fphar.2019.01270 PMC6823301 31708789

[B19] Jung J. Kim H. Y. Kim T. Shin B. H. Park G. S. Park S.. (2012). C-terminal substitution of HBV core proteins with those from DHBV reveals that arginine-rich 167RRRSQSPRR175 domain is critical for HBV replication. PloS One 7, e41087. doi: 10.1371/journal.pone.0041087 22911745 PMC3401125

[B20] Jung J. Kim N. K. Park S. Shin H. J. Hwang S. G. Kim K. (2015). Inhibitory effect of Phyllanthus urinaria L. extract on the replication of lamivudine-resistant hepatitis B virus *in vitro* . BMC Complement Altern. Med. 15, 255. doi: 10.1186/s12906-015-0792-3 26220282 PMC4518506

[B21] Kim J. Kwon H. Kalsoom F. Sajjad M. A. Lee H. W. Lim J. H.. (2022). Ca(2+)/Calmodulin-Dependent Protein Kinase II Inhibits Hepatitis B Virus Replication from cccDNA via AMPK Activation and AKT/mTOR Suppression. Microorganisms 10, 498. doi: 10.3390/microorganisms10030498 35336076 PMC8950817

[B22] Kong F. Li Q. Zhang F. Li X. You H. Pan X.. (2021). Sirtuins as potential therapeutic targets for hepatitis B virus infection. Front. Med. (Lausanne) 8. doi: 10.3389/fmed.2021.751516 PMC8542665 34708060

[B23] Koyuncu E. Budayeva H. G. Miteva Y. V. Ricci D. P. Silhavy T. J. Shenk T.. (2014). Sirtuins are evolutionarily conserved viral restriction factors. mBio 5, e02249-14. doi: 10.1128/mBio.02249-14 25516616 PMC4271551

[B24] Kwon H. Kim J. Song C. Sajjad M. A. Ha J. Jung J.. (2023). Peptidyl-prolyl cis/trans isomerase Pin1 interacts with hepatitis B virus core particle, but not with HBc protein, to promote HBV replication. Front. Cell Infect. Microbiol. 13. doi: 10.3389/fcimb.2023.1195063 PMC10315659 37404723

[B25] Ladner S. K. Otto M. J. Barker C. S. Zaifert K. Wang G. H. Guo J. T.. (1997). Inducible expression of human hepatitis B virus (HBV) in stably transfected hepatoblastoma cells: a novel system for screening potential inhibitors of HBV replication. Antimicrob. Agents Chemother. 41, 1715–1720. doi: 10.1128/aac.41.8.1715 9257747 PMC163991

[B26] Levrero M. Pollicino T. Petersen J. Belloni L. Raimondo G. Dandri M. (2009). Control of cccDNA function in hepatitis B virus infection. J. Hepatol. 51, 581–592. doi: 10.1016/j.jhep.2009.05.022 19616338

[B27] Locatelli M. Quivy J. P. Chapus F. Michelet M. Fresquet J. Maadadi S.. (2022). HIRA supports hepatitis B virus minichromosome establishment and transcriptional activity in infected hepatocytes. Cell Mol. Gastroenterol. Hepatol. 14, 527–551. doi: 10.1016/j.jcmgh.2022.05.007 35643233 PMC9304598

[B28] Lombardo G. E. Russo C. Maugeri A. Navarra M. (2024). Sirtuins as players in the signal transduction of citrus flavonoids. Int. J. Mol. Sci. 25, 1956. doi: 10.3390/ijms25041956 38396635 PMC10889095

[B29] Martinez M. G. Boyd A. Combe E. Testoni B. Zoulim F. (2021). Covalently closed circular DNA: The ultimate therapeutic target for curing HBV infections. J. Hepatol. 75, 706–717. doi: 10.1016/j.jhep.2021.05.013 34051332

[B30] North B. J. Marshall B. L. Borra M. T. Denu J. M. Verdin E. (2003). The human Sir2 ortholog, SIRT2, is an NAD+-dependent tubulin deacetylase. Mol. Cell 11, 437–444. doi: 10.1016/s1097-2765(03)00038-8 12620231

[B31] North B. J. Verdin E. (2007). Interphase nucleo-cytoplasmic shuttling and localization of SIRT2 during mitosis. PloS One 2, e784. doi: 10.1371/journal.pone.0000784 17726514 PMC1949146

[B32] Outeiro T. F. Kontopoulos E. Altmann S. M. Kufareva I. Strathearn K. E. Amore A. M.. (2007). Sirtuin 2 inhibitors rescue alpha-synuclein-mediated toxicity in models of Parkinson’s disease. Science 317, 516–519. doi: 10.1126/science.1143780 17588900

[B33] Piracha Z. Z. Kwon H. Saeed U. Kim J. Jung J. Chwae Y. J.. (2018). Sirtuin 2 isoform 1 enhances hepatitis B virus RNA transcription and DNA synthesis through the AKT/GSK-3β/β-catenin signaling pathway. J. Virol. 92, e00955-18. doi: 10.1128/jvi.00955-18 30111572 PMC6189494

[B34] Piracha Z. Z. Saeed U. Kim J. Kwon H. Chwae Y. J. Lee H. W.. (2020). An alternatively spliced sirtuin 2 isoform 5 inhibits hepatitis B virus replication from cccDNA by repressing epigenetic modifications made by histone lysine methyltransferases. J. Virol. 94, e00926-20. doi: 10.1128/jvi.00926-20 32493816 PMC7394897

[B35] Pollicino T. Belloni L. Raffa G. Pediconi N. Squadrito G. Raimondo G.. (2006). Hepatitis B virus replication is regulated by the acetylation status of hepatitis B virus cccDNA-bound H3 and H4 histones. Gastroenterology 130, 823–837. doi: 10.1053/j.gastro.2006.01.001 16530522

[B36] Ren J. H. Hu J. L. Cheng S. T. Yu H. B. Wong V. K. W. Law B. Y. K.. (2018). SIRT3 restricts hepatitis B virus transcription and replication through epigenetic regulation of covalently closed circular DNA involving suppressor of variegation 3-9 homolog 1 and SET domain containing 1A histone methyltransferases. Hepatology 68, 1260–1276. doi: 10.1002/hep.29912 29624717

[B37] Roche K. L. Remiszewski S. Todd M. J. Kulp J. L. 3rd Tang L. Welsh A. V.. (2023). An allosteric inhibitor of sirtuin 2 deacetylase activity exhibits broad-spectrum antiviral activity. J. Clin. Invest. 133, e158978. doi: 10.1172/jci158978 37317966 PMC10266789

[B38] Saeed U. Kim J. Piracha Z. Z. Kwon H. Jung J. Chwae Y. J.. (2019). Parvulin 14 and Parvulin 17 Bind to HBx and cccDNA and Upregulate Hepatitis B Virus Replication from cccDNA to Virion in an HBx-Dependent Manner. J. Virol. 93, e01840-18. doi: 10.1128/jvi.01840-18 30567987 PMC6401437

[B39] Saeed U. Piracha Z. Z. Kwon H. Kim J. Kalsoom F. Chwae Y. J.. (2021). The HBV core protein and core particle both bind to the PPiase par14 and par17 to enhance their stabilities and HBV replication. Front. Microbiol. 12. doi: 10.3389/fmicb.2021.795047 PMC8713550 34970249

[B40] Silva R. F. E. Bassi G. Câmara N. O. S. Moretti N. S. (2023). Sirtuins: Key pieces in the host response to pathogens’ puzzle. Mol. Immunol. 160, 150–160. doi: 10.1016/j.molimm.2023.06.010 37437515

[B41] Tang L. Remiszewski S. Snedeker A. Chiang L. W. Shenk T. (2024). An allosteric inhibitor of sirtuin 2 blocks hepatitis B virus covalently closed circular DNA establishment and its transcriptional activity. Antiviral Res. 226, 105888. doi: 10.1016/j.antiviral.2024.105888 38641024 PMC12053749

[B42] Vaquero A. Scher M. B. Lee D. H. Sutton A. Cheng H. L. Alt F. W.. (2006). SirT2 is a histone deacetylase with preference for histone H4 Lys 16 during mitosis. Genes Dev. 20, 1256–1261. doi: 10.1101/gad.1412706 16648462 PMC1472900

[B43] Wang F. Song H. Xu F. Xu J. Wang L. Yang F.. (2023). Role of hepatitis B virus non-structural protein HBx on HBV replication, interferon signaling, and hepatocarcinogenesis. Front. Microbiol. 14. doi: 10.3389/fmicb.2023.1322892 PMC10767994 38188582

[B44] Werle-Lapostolle B. Bowden S. Locarnini S. Wursthorn K. Petersen J. Lau G.. (2004). Persistence of cccDNA during the natural history of chronic hepatitis B and decline during adefovir dipivoxil therapy. Gastroenterology 126, 1750–1758. doi: 10.1053/j.gastro.2004.03.018 15188170

[B45] Wu C. S. Chien Y. C. Yen C. J. Wu J. Y. Bai L. Y. Yu Y. L. (2023). EZH2-mediated epigenetic silencing of tumor-suppressive let-7c/miR-99a cluster by hepatitis B virus X antigen enhances hepatocellular carcinoma progression and metastasis. Cancer Cell Int. 23, 199. doi: 10.1186/s12935-023-03002-9 37689710 PMC10493019

[B46] Wu Q. J. Zhang T. N. Chen H. H. Yu X. F. Lv J. L. Liu Y. Y.. (2022). The sirtuin family in health and disease. Signal Transduct Target Ther. 7, 402. doi: 10.1038/s41392-022-01257-8 36581622 PMC9797940

[B47] Yamasaki C. Tateno C. Aratani A. Ohnishi C. Katayama S. Kohashi T.. (2006). Growth and differentiation of colony-forming human hepatocytes *in vitro* . J. Hepatol. 44, 749–757. doi: 10.1016/j.jhep.2005.10.028 16469405

[B48] Yan H. Zhong G. Xu G. He W. Jing Z. Gao Z.. (2012). Sodium taurocholate cotransporting polypeptide is a functional receptor for human hepatitis B and D virus. Elife 1, e00049. doi: 10.7554/eLife.00049 23150796 PMC3485615

[B49] Yang B. Li B. Jia L. Jiang Y. Wang X. Jiang S.. (2020a). 3D landscape of Hepatitis B virus interactions with human chromatins. Cell Discovery 6, 95. doi: 10.1038/s41421-020-00218-1 33372176 PMC7769987

[B50] Yang S. Chen W. Jin S. Luo G. Jing X. Liu Q.. (2022). SUV39H1 regulates corneal epithelial wound healing via H3K9me3-mediated repression of p27. Eye Vis. (Lond) 9, 4. doi: 10.1186/s40662-022-00275-5 35101125 PMC8805298

[B51] Yang W. Chen W. Su H. Li R. Song C. Wang Z.. (2020b). Recent advances in the development of histone deacylase SIRT2 inhibitors. RSC Adv. 10, 37382–37390. doi: 10.1039/d0ra06316a 35521274 PMC9057128

[B52] Yu H. B. Cheng S. T. Ren F. Chen Y. Shi X. F. Wong V. K. W.. (2021). SIRT7 restricts HBV transcription and replication through catalyzing desuccinylation of histone H3 associated with cccDNA minichromosome. Clin. Sci. (Lond) 135, 1505–1522. doi: 10.1042/cs20210392 34128977

[B53] Yu H. B. Jiang H. Cheng S. T. Hu Z. W. Ren J. H. Chen J. (2018). AGK2, A SIRT2 inhibitor, inhibits hepatitis B virus replication *in vitro* and *in vivo* . Int. J. Med. Sci. 15, 1356–1364. doi: 10.7150/ijms.26125 30275764 PMC6158674

[B54] Yuan S. Liao G. Zhang M. Zhu Y. Xiao W. Wang K.. (2021). Multiomics interrogation into HBV (Hepatitis B virus)-host interaction reveals novel coding potential in human genome, and identifies canonical and non-canonical proteins as host restriction factors against HBV. Cell Discovery 7, 105. doi: 10.1038/s41421-021-00337-3 34725333 PMC8560872

