# Supplementary figures and images for: Sirtuin 2 inhibitor AGK2 exerts antiviral effects by inducing epigenetic suppression of hepatitis B virus covalently closed circular DNA through recruitment of repressive histone lysine methyltransferases and reduction of cccDNA
Source: Front Cell Infect Microbiol. 2025 Apr 9;15:1537929. doi: 10.3389/fcimb.2025.1537929 (PMC12014779; doi:10.3389/fcimb.2025.1537929)

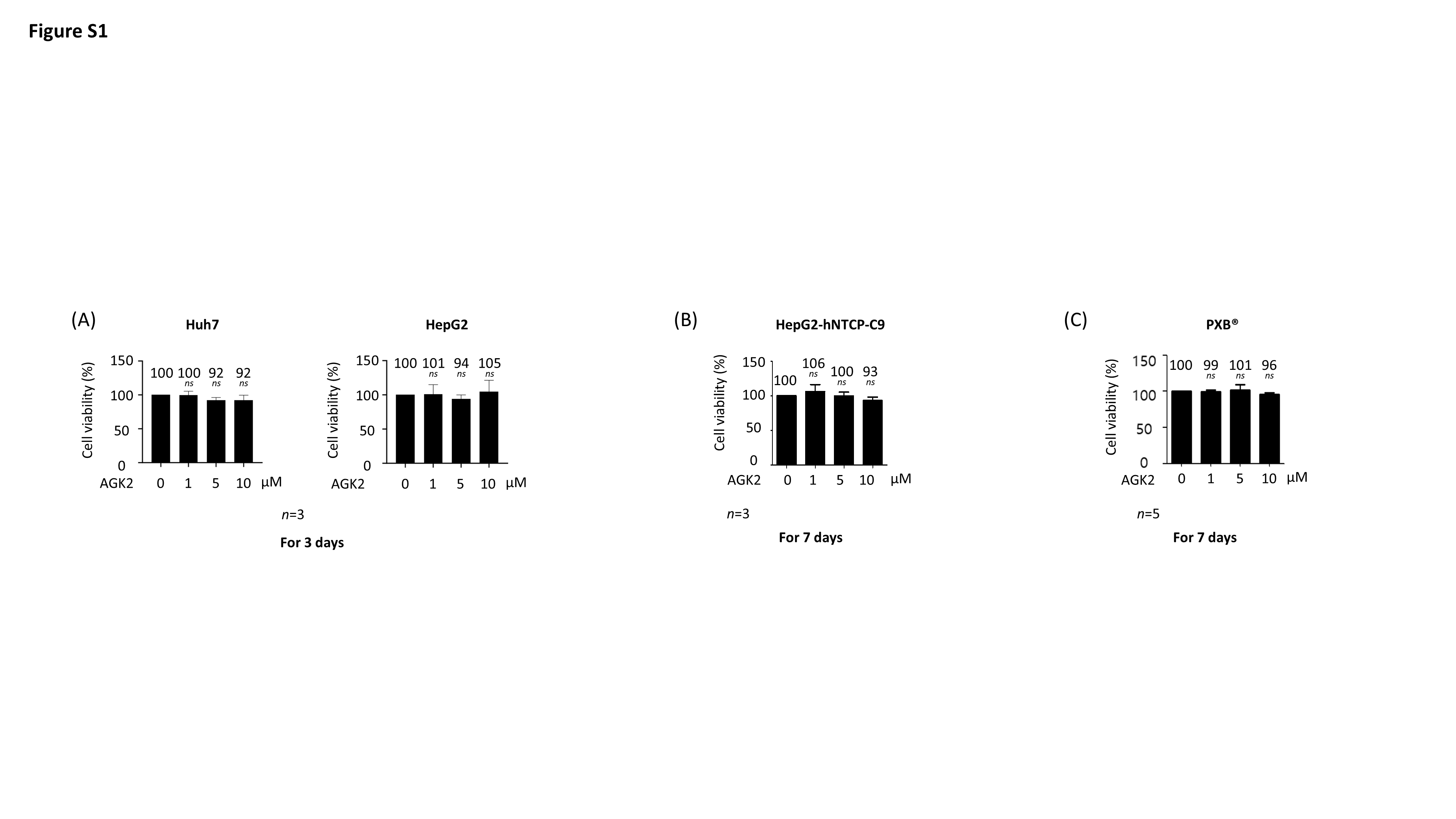

Supplement: Supplementary Figure 1 — AGK2 is not cytotoxic to Huh7, HepG2, HepG2-NTCP-C9, or PXB® cells. (A) At 24 h after seeding, Huh7 and HepG2 cells were treated with AGK2 for 72 h. (B, C) At 24 h after seeding, HepG2-NTCP-C9 and PXB® cells were treated with AGK2 for 7 days. MTT assay (A) or MTS (B, C) assays were performed to measure cell viability. Data from three (A, B) or five (C) independent experiments were analyzed using the ImageJ 1.50b software program. The bars represent the mean ± SD of three (A, B) or five (C) independent experiments. Statistical significance was determined using Student’s t-test. ns, not significant relative to the control. [file Image1.tif]
